# Immunohistochemical Analysis of the Expression of Adhesion Proteins: TNS1, TNS2 and TNS3 in Correlation with Clinicopathological Parameters in Gastric Cancer

**DOI:** 10.3390/biom11050640

**Published:** 2021-04-26

**Authors:** Marcin Nizioł, Justyna Zińczuk, Konrad Zaręba, Katarzyna Guzińska-Ustymowicz, Anna Pryczynicz

**Affiliations:** 1 Department of General Pathomorphology, Medical University of Białystok, Kilińskiego 1, 15-089 Białystok, Poland; marcin.niziol@umb.edu.pl (M.N.); katarzyna.guzinska-ustymowicz@umb.edu.pl (K.G.-U.); 2 Department of Clinical Laboratory Diagnostics, Medical University of Białystok, Kilińskiego 1, 15-089 Białystok, Poland; justyna.zinczuk@umb.edu.pl; 3 2nd Clinical Department of General and Gastroenterological Surgery, Medical University of Białystok, Kilińskiego 1, 15-089 Białystok, Poland; konrad.zareba@umb.edu.pl

**Keywords:** gastric cancer, tensins, adhesion proteins, immunohistochemistry

## Abstract

Tensins belong to the group of adhesion proteins that are involved in cell adhesion and migration, actin cytoskeleton maintenance and intercellular communication. TNS1, TNS2 and TNS3 proteins expression was evaluated in 90 patients with gastric cancer by immunohistochemistry method. TNS1 was more frequently present in non-differentiated tumors compared to poorly and moderately differentiated tumors (*p* = 0.016). TNS1 was also more often observed in metastatic tumors compared to those without distant metastases (*p* = 0.001). TNS2 was more common in moderately differentiated tumors than in poorly or non-differentiated ones (*p* = 0.041). TNS2 expression was also more frequently present in tumors with peritumoral inflammation (*p* = 0.041) and with concomitant *H. pylori* infection (*p* = 0.023). In contrast, TNS3 protein was more prevalent in moderately than in poorly and non-differentiated tumors (*p* = 0.023). No significant relationship was found between tensins’ expression and the overall survival rate of patients. TNS1 protein expression is associated with a poor-prognosis type of GC. Higher expression of TNS2 is accompanied by peritumoral inflammation and *H. pylori* infection, which favor the development of GC of a better prognosis, similarly to higher TNS3 protein expression.

## 1. Introduction

Annually, gastric cancer (GC) contributes to the death of nearly 6% of malignant cancer patients worldwide, which makes it the fifth cause of death caused by various cancers [1]. GC affects men more frequently than women and is more and more often diagnosed in young people (<40 years of age) with a history of this type of cancer in their family [2]. For early-stage GC, the five-year survival rate is approximately 90% [3]. It is therefore reasonable to conduct research aimed at identifying changes in the molecular and biochemical profile specific to the early stages of GC progression. In recent years, numerous studies have focused on adhesion proteins as anchor points in anti-cancer therapies. Adhesion proteins may also play an important role in early cancer detection. One of the protein families involved in tumorigenesis are tensins (TNSs). So far, four proteins (TNS1-4) have been recognized and classified as belonging to this family, based on structural similarity. Typical domains for tensins are SH2 (Src homology 2 domain) and PTB (phosphotyrosine-binding domain). A characteristic feature of tensins is the ability of their SH2 domain to interact with tyrosine residues of the kinases: PI3K (phosphoinositide 3-kinase) and FAK (focal adhesion kinase) [4,5] as well as p130Cas [6]. The PTB domain, in turn, can bind to the β1-, 3-, 5- and 7-integrin tails [7]. Furthermore, TNS1–3 has an ABD (actin-binding domain) at the N-terminal fragment. The presence of PTB and ABD domains enables binding to integrin receptors and actin filaments, thus allowing for the contact between the intracellular environment (cytoskeleton) and the extracellular matrix (ECM). Through these domains, TNS1-3 are able to influence many intracellular signaling pathways, e.g., EGFR, c-Met [8], Rho GAP DLC1 (deleted in liver cancer 1) [9], paxillin [10] and c-Cbl [11]. The importance of tensins 1-3 in cell adhesion and migration has been demonstrated through the presence of these proteins in podosomes [12] and invadopodia [13] that condition the phenomenon of epithelial-mesenchymal transition (EMT). The articles presented herein demonstrate the multitude of tensin-dependent processes, including cell adhesion, migration, maintenance of the actin cytoskeleton and intercellular communication [14].

Literature reports indicate that TNS1 expression is strongly present in the myocardium, kidneys, lungs, small and large intestine, ovaries and prostate [15], while no expression of this protein has been observed in the brain, thymus and leukocytes [16]. TNS1 expression is stimulated, for example, by angiotensin [17], oncogenes (v-Src, BCR-ABL) [18], PDGF [19] or thrombin [20], and is hindered by AMP-activated protein kinase (AMPK). At the cellular level, TNS1, in addition to participating in tensin-specific signaling pathways, is involved in apoptosis as a substrate of the active form of caspase-3 [7]. TNS2, unlike TNS1, interacts with Axl tyrosine residue [21]. The overexpression of this protein promotes apoptosis activation and limits cancer cell proliferation [22] by inhibiting the activity of Akt kinase [17]. TNS3 is structurally deficient in the ABD II (present in TNS1) and C1 (present in TNS2) domains [8]. TNS3 is known to be an adaptor protein in podosome formation by interacting with Dock5 [23]. It is overexpressed in cancer cells, which causes their enhanced invasiveness [24]. It has been observed that increased migration capacity of cancer cells is a result of phosphorylation of the SH2 domain by Src [25]. Silencing the expression of this protein inhibits the growth and migration of cancer cells [25]. It has also been demonstrated that TNS3 expression can be regulated by epigenetic mechanisms [26].

The subject of this study was the immunohistochemical evaluation of the expression of tensins 1-3 in gastric cancer (stages I-IV) as well as the assessment of the correlation of tensin expression with selected clinicopathological parameters and patients’ survival.

## 2. Materials and Methods

### 2.1. Study Group

The study was conducted on a group of 90 patients diagnosed with gastric cancer, who were surgically treated at the 2nd Clinical Department of General and Gastroenterological Surgery, Medical University of Bialystok in the years 2005–2015. Tissue material was obtained from the archives of the Academic Centre for Pathomorphological Diagnostics and Molecular Genetics in Bialystok. Patients were included in the study group based on a diagnosed adenocarcinoma at any stage. The exclusion criteria were: squamous cell carcinoma and other nonepithelial neoplasms, metastases of other neoplasms to the stomach and lack of complete medical documentation. Normal mucosa from the tumor environment was used as the control tissue. The study was approved by the Bioethics Committee of the Medical University of Bialystok, permission no.: R-I-002/29/2019. The study was conducted in accordance with the World Medical Association Declaration of Helsinki for ethical principles for medical research involving human subjects. The characteristics of the study group are presented in Table 1.

### 2.2. Tissue Preparations

Tissue sections taken during the surgery were fixed in 4% buffered formalin solution and embedded in paraffin. The paraffin blocks were then sliced with a microtome into approximately 4-µm-thick slides and stained with hematoxylin and eosin. Via a routine histopathological examination, we assessed the histological type of tumor, histopathological grade (G), stage (pT) and the presence of lymph node metastases (pN), blood and lymphatic vessel infiltration, perineural cancer cells infiltration, peritumoral inflammation and the degree of desmoplasia. Moreover, *H. pylori* infection was assessed in Giemsa-stained preparations. The following information was selected from the histopathological diagnosis sheets: age and gender of the patients, tumor diameter and location, presence of distant metastases and type of cancer according to the Lauren classification.

### 2.3. Immunohistochemistry

Immunohistochemical (IHC) staining was performed on 90 gastric cancer tissues using the polymer method. Paraffin blocks were cut with a microtome into approximately 4-µm-thick sections on silanized slides. The microscope slides were incubated overnight at 60 °C and then deparaffinized in xylene solutions and rehydrated in a series of alcohols of decreasing concentration (2 × 99.9%, 96%, 70%). Antigen retrieval was performed in citric buffer of pH = 6.0 in a water bath at 97.5 °C for 20 min, and then in room temperature for 20 min. Endogenous peroxidase was blocked by using 3% hydrogen peroxide for 10 min. Subsequently, non-specific antibody binding was blocked by using horse serum (anti-mouse/rabbit serum produced in Horse, Vector Laboratories, Eching, Germany) for 20 min. The sections were then incubated with polyclonal anti-TNS1 (clone HPA036089, Sigma-Aldrich, Stockholm, Sweden), anti-TNS2 (clone HPA034659, Sigma Aldrich, Stockholm, Sweden) and anti-TNS3 (clone HPA055338, Sigma-Aldrich, Stockholm, Sweden) polyclonal antibodies at dilutions of: 1:200, 1:100 and 1:250, respectively, for 30 min at room temperature. Antibody binding sites were visualized using the ImmPress Universal Antibody Polymer Reagent kit (Vector Laboratories, Eching, Germany) as well as ImmPACT DAB chromogen (Vector Laboratories, Eching, Germany). Cell nuclei were stained with hematoxylin. Next, the preparations were dehydrated in a series of alcohols of increasing concentration, and washed in xylene solutions.

### 2.4. Validation of TNS1, TNS2, TNS3 Expression Detection

We particularly focused on obtaining reliable results of immunohistochemical staining. In order to optimize the TNS1, TNS2 and TNS3 protein staining procedure, positive and negative controls were performed and selected dilutions of primary antibodies (1:50, 1:75, 1:100, 1:200, 1:250, 1:400), as well as selected incubation times (30 min, 60 min, 120 min) were tested. Antigen retrieval in pH = 6.0 and pH = 9.0 buffers was also performed during the controls.

### 2.5. Microscopic Evaluation

The slides were viewed and evaluated by two independent pathomorphologists on an Olympus BX41 light microscope. The expression of TNS1, TNS2 and TNS3 proteins was assessed at 100× magnification in 10 representative fields of view. In each of them, we evaluated ≥100 tumor cells. Protein expression was observed in both the cell membrane and cytoplasm. Cut-off points were statistically evaluated. The presence of TNS1 protein in >5% of tumor cells was considered positive expression; for TNS2, it was the presence in >10% of tumor cells, and for TNS3, in ≥20% of tumor cells.

### 2.6. Statistical Analysis

The comparison of protein expression between the study groups was performed using Student’s *t*-test. TNS1 expression was compared with the expression of TNS2 protein; TNS1 was compared with TNS3, and TNS2 with TNS3. The comparison of TNS1, TNS2 and TNS3 expressions with the selected clinicopathological parameters was performed by means of the Mann-Whitney U test for two groups, and the Kruskal-Wallis test for three and more groups. Additionally, for the Kruskal-Wallis test, the Dunn’s Multiple Comparison post hoc test was conducted. A value of *p* < 0.05 was considered statistically significant. The overall survival analysis was performed using the Kaplan–Meier estimate. For the analysis, the Statistica 13 program (Statsoft, Krakow, Poland) was used. Missing data were eliminated in pairs.

## 3. Results

### 3.1. Expression of TNS1, TNS2 and TNS3 in Gastric Cancer Samples

**Figure 1:**
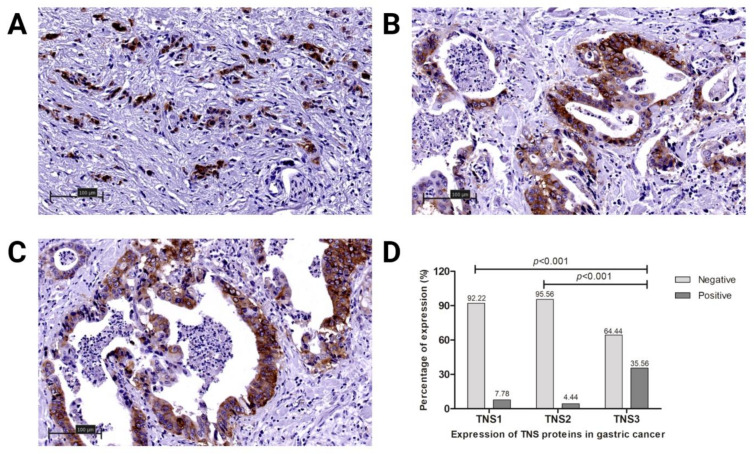
Tensin (TNS) protein expression levels in gastric cancer (GC): (**A**) TNS1 in non-differentiated GC, magnification, ×200; (**B**) TNS2 in moderately differentiated GC, magnification, ×200; (**C**) TNS3 in moderately differentiated GC, magnification, ×200; (**D**) comparison of TNS1-3 in GC. Statistical analysis performed using Student’s *t*-test.

The expression of TNS1, TNS2 and TNS3 was examined immunohistochemically in 90 gastric cancer samples and 20 normal gastric tissues. In all cases of control group in normal gastric mucosa, expression of these proteins was absent. The expression of the tensins was higher in cancer cells compared to normal gastric mucosa cells. A microscopic analysis demonstrated that positive expression of TNS1, TNS2 and TNS3 proteins in tumor cells was present in 7 (7.78%), 4 (4.44%) and 32 (35.56%) out of 90 patients, respectively. In tumor cells of all cases, the expression of these proteins was observed in the cell membrane and cytoplasm (Figure 1A–D).

### 3.2. Comparison of TNS1, TNS2 and TNS3 Expression with Clinicopathological Parameters of GC

**Figure 2:**
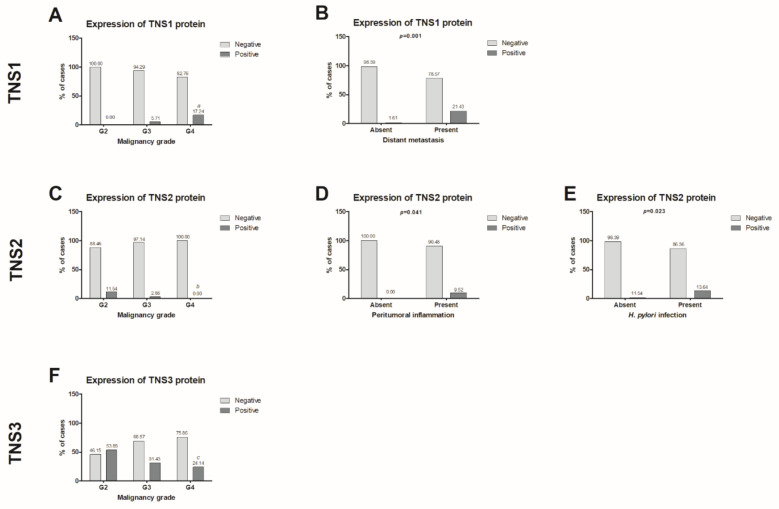
Comparison of TNS1-3 expression levels with the selected clinicopathological parameters: (**A**) TNS1 and malignancy grade, G2 vs. G4 statistically significant at *p* = 0.02; (**B**) TNS1 and distant metastasis; (**C**) TNS2 and malignancy grade, G2 vs. G4 statistically significant at *p* = 0.045; (**D**) TNS2 and peritumoral inflammation; (**E**) TNS2 and *H. pylori* infection; (**F**) TNS3 and malignancy grade, G2 vs. G4 statistically significant at *p* = 0.025. The comparison of TNS1-3 expressions with the selected clinicopathological parameters was performed using the Mann-Whitney U test for two groups, and the Kruskal-Wallis test for 3 and more groups. Dunn’s Multiple Comparison post hoc test was applied for Kruskal-Wallis test. A value of *p* < 0.05 was considered statistically significant. *^a^*^, *b*, *c*^ *p* < 0.05 G2 vs. G4 TNS1-3 expression levels in gastric cancer. G2—moderately differentiated GC, G3—poorly differentiated GC, G4—non-differentiated GC.

The statistical analysis demonstrated a significant correlation between TNS1 expression and the malignancy grade (Figure 2A). The study group consisted of 28.89% moderately differentiated, 38.89% poorly differentiated and 32.22% non-differentiated cancers. TNS1 protein was more frequently present in non-differentiated tumors (17.24% of patients) than in poorly differentiated (5.71% of patients) and moderately differentiated (0.00% of patients) cancers (*p* = 0.016). The statistical analysis also showed that TNS1 protein expression correlates with the presence of distant organ metastases (Figure 2B). In the study group, 68.89% of tumors did not metastasize to distant tissues and 31.11% of tumors showed such metastases. TNS1 expression was more frequently observed in metastatic tumors (21.43% of patients) compared to cancers without distant metastases (1.61% of patients) (*p* = 0.001). The results are presented in Table 2.

A correlation was demonstrated between TNS2 expression and the malignancy grade (Figure 2C). TNS2 was more frequently observed in moderately differentiated tumors (11.54% of patients) as compared to poorly differentiated (2.86% of patients) and non-differentiated tumors (0.00% of patients) (*p* = 0.041). There was also a statistically significant relationship between TNS2 expression and peritumoral inflammation (Figure 2D). In the study group, there were 50% of tumors without inflammation and the same number with peritumoral inflammation. TNS2 expression occurred more frequently in cancers with peritumoral inflammation (9.52% of patients) compared to tumors without inflammation in the surrounding tissues (0.00% of patients) (*p* = 0.041). TNS2 protein expression was also shown to correlate with *H. pylori* infection (Figure 2E). The study group comprised 73.81% of cancers without *H. pylori* infection and 26.19% of tumors accompanied by this infection. TNS2 expression was more frequently observed in tumors with *H. pylori* infection (13.64% of patients) than in cases without the infection (1.61% of patients) (*p* = 0.023). The results are presented in Table 3.

The statistical analysis revealed a correlation between TNS3 expression and the malignancy grade (Figure 2F). TNS3 protein was more frequently present in moderately differentiated tumors (53.85% of patients) than in poorly differentiated (31.43% of patients) and non-differentiated tumors (24.14% of patients) (*p* = 0.023). The results are presented in Table 4.

### 3.3. Analysis of the Correlation of TNS1, TNS2 and TNS3 Expression Levels with the Overall Survival of Patients

**Figure 3:**
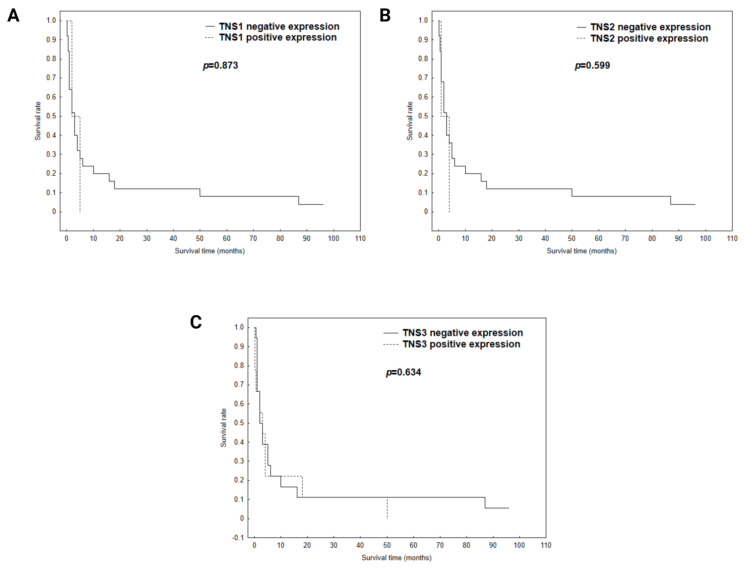
Total survival analysis performed using a Kaplan-Meier test of tensins in gastric cancer (**A**) TNS1; (**B**) TNS2; (**C**) TNS3.

The statistical analysis showed no significant correlation between TNS1, TNS2 and TNS3 expressions and the overall survival of patients (*p* = 0.873, *p* = 0.599, *p* = 0.634, respectively) (Figure 3A–C).

## 4. Discussion

Gastric cancer belongs to common malignant tumors and is a cause of death of a high number of patients. Therefore, it poses a challenge to scientists studying its pathomechanisms and searching for specific and sensitive markers of tumor cell formation that would enable early detection of cancerous lesions. Another important goal of scientists and clinicians is the search for new therapeutic targets that would increase the effectiveness of anti-cancer therapies and improve the quality of life of GC patients. From the perspective of cancer progression, the key ability of cancer cells is to migrate and invade, thus leading to the formation of distant metastases. Proteins involved in actin cytoskeleton remodeling are factors that enhance the migratory potential of cancer cells. Of the numerous types of adhesion proteins, tensins are known to enable the interaction between the intracellular environment and extracellular matrix.

The aim of our study was to evaluate the immunohistochemical expression of proteins from the tensin family: TNS1-3 in patients diagnosed with stages I-IV of gastric cancer, and then to analyze the correlation between protein expression levels and selected clinicopathological parameters. In tumor cells of all cases, we observed both membranous and cytoplasmic expressions of TNS1, TNS2, and TNS3 proteins. No correlations between immunostaining intensity and the cellular location of these proteins were found in the literature. We noted that the positive expression of TNS1 and TNS2 was not common in tumor cells, whereas the positive expression of TNS3 occurred in tumor cells of about one-third of GC patients. The observed dependencies may result from differences in the expression pattern of TNS1-3. TNS1, similarly to TNS2, is expressed in the heart, kidneys, skeletal muscles and liver [15].

Our study demonstrated that the positive expression of TNS1-3 proteins correlated with the malignancy grade of GC. TNS1 expression was over three times more frequent in non-differentiated gastric tumors than in the poorly differentiated type, whereas no TNS1 was detected in moderately differentiated tumors. Although there are no reports in the literature indicating a role of TNS1 in the development of non-differentiated cancer, we hypothesized that this protein may be overexpressed in intensely growing tissues. TNS1 is known to be essential for the formation of fibrils in extracellular vesicles [27] and myofibroblast differentiation [28] during embryogenesis, while the absence of TNS1 leads, inter alia, to kidney degeneration [29]. In contrast, positive expression of TNS2 and TNS3 was observed more frequently in moderately differentiated gastric cancer than in poorly or undifferentiated tumors. It is known that the degree of differentiation of glandular ducts in the stomach enables to determine the disease progression. Higher GC cell differentiation is related with better patient’s prognosis [30]. According to the results of our research, the studied proteins may be involved in the process of differentiation of gastric cancer cells, although their role varies depending on the tensin type. The available literature provides no reports confirming a significant correlation between the expression level of these proteins and the degree of tumor malignancy, which underlines the uniqueness of our study. TNS1 expression is more common in non-differentiated GC associated with a worse prognosis.

According to our further observations, positive TNS1 expression is correlated with the presence of distant organ metastases, which supports the hypothesis that TNS1 may play an important role as a prognostic factor for GC. In primary prostate cancer, Zhu et al. [31] observed a similar relationship between TNS1 mRNA expression levels and tumor metastases to bones in which the expression of this gene in metastatic cells was significantly higher. Moreover, Martuszewska et al. [32] suggested that the presence of TNS3 at the cell periphery stabilizes the structure of the cells by limiting their motility, and thus reduces metastatic potential. An RNA-seq analysis revealed a significant involvement of TNS2 in the formation of focal adhesions which are associated with the development of metastases in numerous cancers [33]. Our findings did not show any relationships between TNS2 and TNS3 expression and metastases.

In our study, we observed statistically significant differences in TNS2 expression between cancers with and without a peritumoral inflammation. Moreover, we noted a significant correlation between this protein expression and the concomitant *H. pylori* infection. The tumor microenvironment is diverse in terms of cell types. Tumor cells are often accompanied by inflammatory cells whose role in tumorigenesis remains unclear: they may have either pro- or anti-cancer effects [34]. An immune response should lead to the recognition and destruction of cancer cell clones; however, in many tumors, we observed disturbances in the functioning of the immune system. This is probably due to the defense mechanisms of the developing tumor, i.e., the presence of poorly immunogenic antigens, as well as disturbances in antigen presentation by inflammatory cells, or production of immunosuppressive factors by cancer cells [35]. It is probable that the observed positive TNS2 expression with simultaneous peritumoral inflammation is related to *H. pylori* infection. Numerous malignancies are known to develop at sites of infection and ongoing inflammation [34]. Since we observed a correlation between higher TNS2 expression in moderately differentiated GC with peritumoral inflammation and *H. pylori* infection, further studies are required to confirm the existence of such a relationship.

What is more, we did not demonstrate a statistically significant correlation between TNS1-3 expression levels and the survival of patients. However, this absence of statistical significance may result from the low number of subjects in our study group as well as the lack of available survival data in the case of some patients. These limitations of the study prevented us from drawing conclusions about the relationship of TNS1-3 with the survival rate of gastric cancer patients. Zhan et al. [36] observed that TNS1 overexpression correlates with prolonged metastasis-free survival, whereas in metastatic breast cancer, the expression of this protein is significantly reduced. In contrast, Zhang et al. [37] demonstrated that TNS1 expression levels and the overall survival of bladder cancer patients were negatively correlated. According to other studies, reduced TNS2 expression is positively correlated with short relapse-free survival of breast and lung cancer patients [38]. It was also shown that patients with kidney cancer and TNS3 detected in their cell membrane prognosed better survival than those lacking TNS3 or with TNS3 present only in the cytoplasm [32].

## 5. Conclusions

In conclusion, our study suggests that TNS1 expression is associated with a GC type of a poorer prognosis and with the occurrence of distant metastases. In contrast, higher TNS2 expression is accompanied by peritumoral inflammation as well as *H. pylori* infection, which favor GC with a better prognosis, as does higher expression of TNS3 protein.

## Figures and Tables

**Table 1:** The characteristics of study group.

Parameter	Number of Cases (%)
Age	
<60	29 (32.22%)
≥60	61 (67.78%)
Gender	
Female	30 (33.33%)
Male	60 (66.67%)
Tumor diameter	
<5 cm	19 (21.11%)
≥5 cm	71 (78.89%)
Tumor localization	
Upper 1/3	17 (18.89%)
Middle1/3	34 (37.78%)
Lower 1/3	16 (17.78%)
Whole stomach	23 (25.55%)
Histological type	
Adenocarcinoma	55 (61.11%)
Adenocarcinoma mucinosum	35 (38.89%)
Histological differentiation	
Moderately differentiated	26 (28.89%)
Poorly differentiated	35 (38.89%)
Non-differentiated	29 (32.22%)
Depth of invasion	
T1	7 (7.78%)
T2	7 (7.78%)
T3	66 (73.33%)
T4	10 (11.11%)
Lymph node metastasis	
Absent	18 (20.00%)
Present	72 (80.00%)
Distant metastasis	
Absent	62 (68.89%)
Present	28 (31.11%)
Blood vessel infiltration	
Absent	49 (84.48%)
Present	9 (15.52%)
Lymphatic vessel infiltration	
Absent	22 (32.84%)
Present	45 (61.16%)
Perineural cancer cells infiltration	
Absent	29 (34.94%)
Present	54 (65.06%)
Peritumoral inflammation	
Absent	42 (50.00%)
Present	42 (50.00%)
Desmoplasia	
Small	56 (66.67%)
Diffuse	28 (33.33%)
*H. pylori* infection	
Absent	62 (73.81%)
Present	22 (26.19%)
Lauren’s classification	
Intestinal	47 (56.63%)
Diffuse	36 (43.37%)

**Table 2:** Correlation between TNS1 protein expression level in gastric cancer and clinicopathological parameters.

Parameter	Expression of TNS1 Protein		*p*-Value *^a^*
	Negative	Positive	
Age			0.296
<60	28 (96.55%)	1 (3.45%)	
≥60	55 (90.16%)	6 (9.84%)	
Gender			0.271
Female	29 (96.97%)	1 (3.33%)	
Male	54 (90.00%)	6 (10.00%)	
Tumor diameter			0.158
<5cm	19 (100%)	0 (0.00%)	
≥5cm	64 (90.14%)	7 (9.86%)	
Tumor localization			0.201
Upper 1/3	16 (94.12%)	1 (5.88%)	
Middle1/3	32 (94.12%)	2 (5.88%)	
Lower 1/3	16 (100%)	0 (0.00%)	
Whole stomach	19 (82.61%)	4 (17.39%)	
Histological type			0.067
Adenocarcinoma	53 (96.36%)	2 (3.64%)	
Adenocarcinoma mucinosum	30 (85.71%)	5 (14.29%)	
Histological differentiation			0.02
Moderately differentiated	26 (100.00%)	0 (0.00%)	(0.016)
Poorly differentiated	33 (94.29%)	2 (5.71%)	
Non-differentiated	24 (82.76%)	5 (17.24%)	
Depth of invasion			0.300
T1	7 (100%)	0 (0.00%)	
T2	7 (100%)	0 (0.00%)	
T3	60 (90.91%)	6 (9.09%)	
T4	9 (90.00%)	1 (10.00%)	
Lymph node metastasis			0.172
Absent	18 (100%)	0 (0.00%)	
Present	65 (90.28%)	7 (9.72%)	
Distant metastasis			0.001
Absent	61 (98.39%)	1 (1.61%)	
Present	22 (78.57%)	6 (21.43%)	
Blood vessel infiltration			0.546
Absent	47 (95.92%)	2 (4.08%)	
Present	9 (100%)	0 (0.00%)	
Lymphatic vessel infiltration			0.107
Absent	22 (100%)	0 (0.00%)	
Present	40 (88.89%)	5 (11.11%)	
Perineural cancer cells infiltration			0.064
Absent	29 (100.00%)	0 (0.00%)	
Present	48 (88.89%)	6 (11.11%)	
Peritumoral inflammation			0.403
Absent	38 (90.48%)	4 (9.52%)	
Present	40 (95.24%)	2 (4.76%)	
Desmoplasia			1.000
Small	52 (92.86%)	4 (7.14%)	
Large	26 (92.86%)	2 (7.14%)	
*H. pylori* infection			0.133
Absent	56 (90.32%)	6 (9.68%)	
Present	22 (100.00%)	0 (0.00%)	
Lauren’s classification			0.260
Intestinal	43 (95.56%)	4 (4.44%)	
Diffuse	32 (88.89%)	4 (11.11%)	

***^a^*** in brackets are *p*-values before Dunn’s Multiple Comparison post hoc test.

**Table 3:** Correlation between TNS2 protein expression level in gastric cancer and clinicopathological parameters.

Parameter	Expression of TNS2 Protein		*p*-Value *^a^*
	Negative	Positive	
Age			0.755
<60	28 (96.55%)	1 (3.45%)	
≥60	58 (95.08%)	3 (4.92%)	
Gender			0.475
Female	28 (93.33%)	2 (6.67%)	
Male	58 (96.67%)	2 (3.33%)	
Tumor diameter			0.848
<5 cm	18 (94.74%)	1 (5.26%)	
≥5 cm	68 (95.77%)	3 (4.23%)	
Tumor localization			0.636
Upper 1/3	17 (100.00%)	0 (0.00%)	
Middle1/3	32 (94.12%)	2 (5.88%)	
Lower 1/3	15 (93.75%)	1 (6.25%)	
Whole stomach	22 (95.65%)	1 (4.35%)	
Histological type			0.105
Adenocarcinoma	51 (92.73%)	4 (7.27%)	
Adenocarcinoma mucinosum	35 (100.00%)	0 (0.00%)	
Histological differentiation			0.045(0.041)
Moderately differentiated	23 (88.46%)	3 (11.54%)	
Poorly differentiated	34 (97.14%)	1 (2.86%)	
Non-differentiated	29 (100.00%)	0 (0.00%)	
Depth of invasion			0.272
T1	6 (85.71%)	1 (14.29%)	
T2	7 (100%)	0 (0.00%)	
T3	63 (95.45%)	3 (4.55%)	
T4	10 (100.00%)	0 (0.00%)	
Lymph node metastasis			0.801
Absent	17 (94.44%)	1 (5.56%)	
Present	69 (95.83%)	3 (4.17%)	
Distant metastasis			0.173
Absent	58 (93.55%)	4 (6.45%)	
Present	28 (100%)	0 (0.00%)	
Blood vessel infiltration			0.546
Absent	47 (95.92%)	2 (4.08%)	
Present	9 (100%)	0 (0.00%)	
Lymphatic vessel infiltration			0.208
Absent	20 (90.91%)	2 (9.09%)	
Present	44 (97.78%)	1 (2.22%)	
Perineural cancer cells infiltration			0.087
Absent	26 (89.66%)	3 (10.34%)	
Present	53 (98.15%)	1 (1.851%)	
Peritumoral inflammation			0.041
Absent	42 (100.00%)	0 (0.00%)	
Present	38 (90.48%)	4 (9.52%)	
Desmoplasia			0.721
Small	53 (94.64%)	3 (5.36%)	
Large	27 (96.43%)	1 (3.57%)	
*H. pylori* infection			0.023
Absent	61 (98.39%)	1 (1.61%)	
Present	19 (86.36%)	3 (13.64%)	
Lauren’s classification			0.697
Intestinal	43 (95.56%)	2 (4.44%)	
Diffuse	35 (97.22%)	1 (2.77%)	

***^a^*** in brackets are *p*-values before Dunn’s Multiple Comparison post hoc test.

**Table 4:** Correlation between TNS3 protein expression level in gastric cancer and clinicopathological parameters.

Parameter	Expression of TNS3 Protein		*p*-Value *^a^*
	Negative	Positive	
Age			0.885
<60	19 (65.52%)	10 (34.48%)	
≥60	39 (63.93%)	22 (36.07%)	
Gender			0.878
Female	19 (63.33%)	11 (36.67%)	
Male	39 (65.00%)	21 (35.00%)	
Tumor diameter			0.897
<5 cm	12 (63.16%)	7 (36.84%)	
≥5 cm	46 (64.79%)	25 (35.21%)	
Tumor localization			0.308
Upper 1/3	13 (76.47%)	4 (23.53%)	
Middle 1/3	22 (64.71%)	12 (35.29%)	
Lower 1/3	9 (56.25%)	7 (43.75%)	
Whole stomach	14 (60.87%)	9 (39.13%)	
Histological type			0.520
Adenocarcinoma	34 (61.82%)	21 (38.18%)	
Adenocarcinoma mucinosum	24 (68.57%)	11 (31.43%)	
Histological differentiation			0.025
Moderately differentiated	12 (46.15%)	14 (53.85%)	(0.023)
Poorly differentiated	24 (68.57%)	11 (31.47%)	
Non-differentiated	22 (75.86%)	7 (24.14%)	
Depth of invasion			0.514
T1	5 (71.43%)	2 (28.57%)	
T2	3 (42.86%)	4 (57.14%)	
T3	42 (63.64%)	24 (36.36%)	
T4	8 (80.00%)	2 (20.00%)	
Lymph node metastasis			0.447
Present	45 (62.50%)	5 (27.78%)	
Absent	13 (72.22%)	27 (37.50%)	
Distant metastasis			0.654
Absent	39 (62.90%)	23 (37.10%)	
Present	19 (67.86%)	9 (32.14%)	
Blood vessel infiltration			0.584
Absent	32 (65.31%)	17 (34.69%)	
Present	5 (55.56%)	4 (44.44%)	
Lymphatic vessel infiltration			0.912
Absent	14 (63.64%)	8 (36.36%)	
Present	28 (62.22%)	17 (37.78%)	
Perineural cancer cells infiltration			0.473
Absent	17 (58.62%)	12 (41.38%)	
Present	36 (66.67%)	18 (33.33%)	
Peritumoral inflammation			0.824
Absent	27 (64.29%)	15 (35.71%)	
Present	26 (61.90%)	16 (38.10%)	
Desmoplasia			0.268
Small	33 (58.93%)	23 (41.07%)	
Large	20 (71.43%)	8 (28.57%)	
*H. pylori* infection			0.952
Absent	39 (62.90%)	23 (37.10%)	
Present	14 (63.64%)	8 (36.36%)	
Lauren’s classification			0.420
Intestinal	26 (57.78%)	19 (42.22%)	
Diffuse	24 (66.67%)	12 (33.33%)	

***^a^*** in brackets are *p*-values before Dunn’s Multiple Comparison post hoc test.

## Data Availability

Data supporting reported results can be obtained from the corresponding author upon request.
